# Spontaneous Hall effect in the Weyl semimetal candidate of all-in all-out pyrochlore iridate

**DOI:** 10.1038/s41467-018-05530-9

**Published:** 2018-08-02

**Authors:** Kentaro Ueda, Ryoma Kaneko, Hiroaki Ishizuka, Jun Fujioka, Naoto Nagaosa, Yoshinori Tokura

**Affiliations:** 1 Department of Applied Physics, University of Tokyo, Tokyo, 113-8656 Japan; 2 PRESTO, Japan Science and Technology Agency, Kawaguchi, Saitama 332-0012 Japan; 3 RIKEN Center for Emergent Matter Science (CEMS), Wako, 351-0198 Japan

## Abstract

Topological quantum states of matter, characterized by geometrical features of electronic band structures, have been extensively studied. Among them, the topological electronic state with magnetic order remains elusive because of a scarce number of examples. Here we present experimental observations proving that the pyrochlore iridate, when electronically tuned, can be a topological Weyl semimetal as predicted by recent theories. We observe a sizable spontaneous Hall conductivity with minimal magnetization only within a few Kelvin below the all-in all-out magnetic ordering temperature. Our theoretical calculation, which is quantitatively consistent with the observation, suggests that the presence of linearly-dispersing crossing points (Weyl points), acting as a source/sink of a quantized magnetic flux, potentially gives rise to such an enormous effect. The manifestation of the salient Hall response provides one important example of topological states, which promotes a better understanding of Weyl semimetal and indicates the new research direction for the topological-materials design.

## Abstract (web summary)

Material with both topological state and magnetic order remains rare. Here, Ueda et al. observe a sizable spontaneous Hall conductivity with minimal magnetization at a few Kelvin below the all-in-all-out magnetic ordering temperature in the predicted magnetic Weyl semimetal *R*_2_Ir_2_O_7_.

## Introduction

Origins of anomalous Hall effect (AHE), conventionally produced by the presence of the magnetization, have been a longstanding issue in condensed matter physics since its discovery more than a century ago^1^. An intrinsic mechanism of AHE was first proposed by Karplus and Luttinger who attributed it to the electronic band structure with spin–orbit interaction^2^, which generates an additional contribution in a Hall current with no energy consumption. Recently, this mechanism has been reformulated in terms of the Berry curvature, i.e., the quantum geometric/topological property of the Bloch wave functions^3^. Since the concept of the topological nature was appreciated, the intrinsic mechanism has received a renewed interest from a broader perspective over the past few decades; the Berry phase is now considered as the key concept for AHE, successfully explaining AHEs observed in a number of magnetic materials^4–9^. Recently, Weyl semimetal (WSM) has drawn much attention as a unique class of materials that potentially shows an enormous Hall response^10,11^. WSM is the semimetal or zero-gap semiconductor in which the two non-degenerate bands cross linearly at the band-touching points, i.e., Weyl points (WPs)^10–13^. Intriguingly, WP can be regarded as a magnetic monopole of Berry curvature in *k*-space, and therefore, its position is expected to be manifested in AHE.

**Fig. 1:**
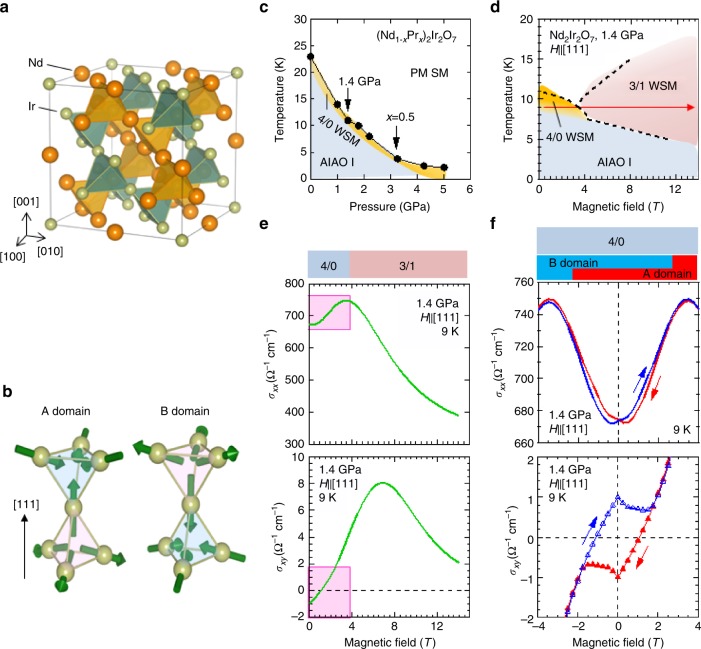
Phase diagram and representative magnetic transport properties in *R*_2_Ir_2_O_7_. **a** Pyrochlore lattice structure. Orange balls depict Nd ions and green ones are Ir ions. **b** Schematic magnetic configuration of all-in all-out state for A domain (left) and B domain (right). Phase diagram of (Nd_1-*x*_Pr_*x*_)_2_Ir_2_O_7_ in the plane of **c** temperature and external pressure, **d** temperature and magnetic field at 1.8 GPa. According to the previous study^22^, the chemical substitution of Nd ions with Pr ones effectively scales with the external pressure *P*, i.e., “chemical pressure”. In Fig. 1c, we apply pressure on *R* = Nd while the *x* = 0.5 composition under ambient pressure corresponds to *P* = 3.3 GPa. PM SM stands for paramagnetic semimetal, 4/0 WSM stands for 4-in 0-out (all-in all-out) Weyl semimetal, AIAO I for all-in all-out insulator, 3/1 WSM for 3-in 1-out Weyl semimetal, respectively. **e** Magnetic field dependence of Hall conductivity and longitudinal conductivity. **f** Magnified figures of Fig. 1e in the field range between −4 T and 4 T. Red and blue marks and lines are on field-decreasing process and on field-increasing process, respectively

A family of pyrochlore iridates *R*_2_Ir_2_O_7_ is the first existing compound that is proposed to realize a magnetic WSM by a first-principle calculation^10^. The pyrochlore iridates host symmetry identical to the diamond lattice (Fig.1a) and are considered to be a fertile ground to potentially produce topologically-nontrivial electronic states^14^. Furthermore, the magnetic ordering configuration shows the all-in all-out (AIAO, 4/0) state which breaks the time-reversal symmetry without reducing the cubic lattice symmetry (Fig. 1b). These conditions are remarkably suitable for the realization of topological states. The recent angle-resolved photoemission (ARPES) study has revealed that the ground state of the paramagnetic metal *R* = Pr is a unique semimetal with a quadratic-band-touching point right across the Fermi level, which evolves into abundant topologically-nontrivial phases by symmetry-breaking perturbations^15,16^. When the time-reversal symmetry is broken by the AIAO magnetic order, for instance, WSM is predicted to emerge with 8 WPs for intermediate electron-correlation strengths^12,13^. In general, WSM is stable against perturbations as each WP is protected by the topological charge. In fact, the *R* = Nd compound undergoes the AIAO magnetic order below *T*_N_, accompanying a metal-insulator transition (Fig. 1c); the theories^10,12,13^ predict the AIAO WSM state in the vicinity of the AIAO charge-gapped state. Nonetheless, the experimental confirmation of WSM under zero magnetic field turns out to be challenging because the charge gap appears to open so easily by the pair annihilation of WPs which quickly immigrate as a function of the magnetic order parameter and collide with each other at the zone boundaries, consequently leaving metallic fragments only in magnetic domain walls as remnants of the surface state in the gapped state^13,17,18^. Therefore, WSM is expected in an extremely narrow temperature region right below *T*_N_, still being missed so far^19^.

Here we exploit the electronic transport measurements on pyrochlore Nd_2_Ir_2_O_7_ and (Nd_0.5_Pr_0.5_)_2_Ir_2_O_7_ by applying pressure and magnetic field to search for the smoking gun of the predicted WSM state. A salient spontaneous Hall effect accompanied by a vanishingly small magnetization is observed only within the narrow temperature window right below *T*_N_. Assuming that the observed Hall effect is provoked by the emergence of WPs which can be regarded as a source or sink of the quantized magnetic flux in *k*-space, we carry out the numerical analysis of the Hall conductivity. As a result, the minimal tilting of the magnetic moment deviating from the ideal AIAO state is proved to cause such an enormous Hall effect. The qualitative consistency with the experiment indicates that the WSM can be realized in pyrochlore iridates, offering a significant step towards the material design for magnetic topological systems.

## Results

### Phase diagram for pyrochlore iridates and spontaneous hall effect

To explore the WPs in this system, we take advantage of the Hall effect which is sensitive to the Berry phase^3^. For this purpose, high-quality single crystals of *R* = Nd (Nd_2_Ir_2_O_7_) were prepared for transport measurements under fine control of temperature, hydrostatic pressure, and magnetic field. Figure 1d shows the phase diagram as a function of magnetic field for Nd_2_Ir_2_O_7_ (*R* = Nd) at the pressure *P* = 1.4 GPa. At zero magnetic field, the magnetic transition occurs at 12 K from paramagnetic to AIAO state. The application of the field along [111] crystalline direction modulates the AIAO pattern by flipping one of four magnetic moments on vertices of a tetrahedron, referred to as 3-in 1-out (3/1) configuration. In accordance, the electronic transport properties change dramatically (Fig. 1e); as the field increases, the longitudinal conductivity *σ*_*xx*_ rises up, reaches the maximum around 4T, and then decreases down to almost a half of its maximum at 14T. Accordingly, the Hall conductivity *σ*_*xy*_ also shows nonmonotonous field dependence (Fig. 1e); as the magnetic field increases, *σ*_*xy*_ increases in a low-field region, reaches the maximum around 7T, and then decreases. According to the ref. ^20^., the magnetization monotonically increases and saturates the value which is expected for Nd-4*f* 3-in 1-out state. The observed *σ*_*xy*_ is different from the conventional AHE which is proportional to magnetization; it presumably reflects the variation of the electronic structures into the other topological state (WSM (3/1)) that can induce the nontrivial contribution to AHE inherent to WPs, as argued in previous reports^20,21^. Hereafter, we shall take an attentive look into the low-field range in which the AIAO order is preferred. Figure 1f shows the magnified view of the low-field region highlighted in Fig. 1e. The *σ*_*xx*_ on the field- decreasing process (denoted by the red line) is smaller than that on the field-increasing process (the blue line) in the positive field region, and they turn opposite in the negative field. This hysteresis can be explained by the unique configuration of AIAO order; there are two types of magnetic domains in AIAO state as depicted in Fig. 1b (here we term A and B domain, respectively), each of which can be aligned by the application of the field along [111] or the opposite direction^22^. The aligned AIAO configuration can induce the odd component of *σ*_*xx*_ as demonstrated in a previous study^23^. Therefore, the butterfly-type hysteresis of *σ*_*xx*_ within ±0 T indicates that the single domain state is realized after applying the magnetic field along [111] direction. Significantly, the hysteresis is also discerned in *σ*_*xy*_; as the magnetic field decreases, *σ*_*xy*_ denoted in red marks decreases towards −1 Ω^−1^ cm^−1^ with a sharp dip at 0 T. *σ*_*xy*_ on the field-increasing process (blue marks) shows the similar field dependency with the opposite sign, shaping an unambiguous diamond-type hysteresis within ±2 T.

**Fig. 2:**
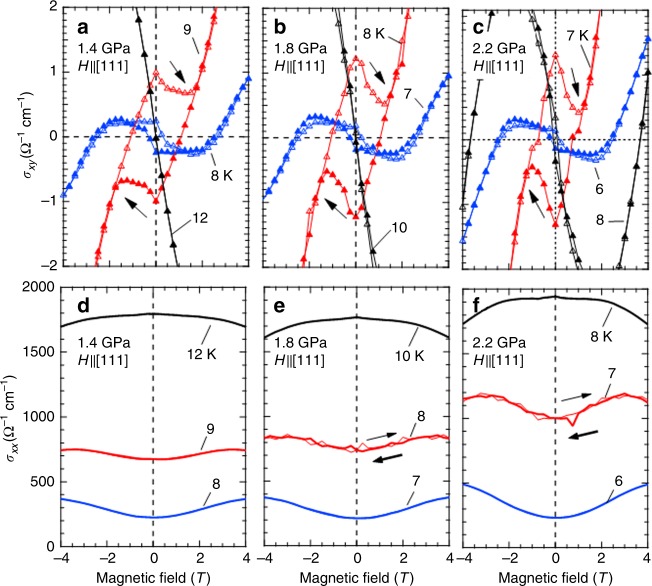
Magnetotransport properties of Nd_2_Ir_2_O_7_ near the transition temperature at several pressures. Magnetic field dependence of Hall conductivity (**a**–**c**) and longitudinal conductivity (**d**–**f**) for a field along the [111] crystallographic direction at **a**, **d** 1.4 GPa, **b**, **e** 1.8 GPa, **c**, **f** 2.2 GPa, respectively. The open (colored) marks are Hall conductivities on increasing (decreasing) field process which is indicated by black arrows

To gain more insight into the spontaneous component of *σ*_*xy*_, we study the temperature and pressure dependence which allows us to control the magnetic state via tuning of the effective bandwidth. As shown in Fig. 1c, the *T*_N_ systematically decreases with increasing hydrostatic pressure due to the enhancement of electron hopping interaction. The top figures of Fig. 2 display the field dependence of *σ*_*xy*_ at several temperatures and pressures. At *T*_N_, where the data are denoted by black marks, *σ*_*xy*_ shows no difference between increasing and decreasing field process. At a lower temperature (denoted in red marks), by contrast, the hysteresis conspicuously shows up with the value of ~±1 Ω^−1^ cm^−1^ at 0 T. On further decreasing temperature, the hysteresis loop is abruptly closed and the spontaneous component of *σ*_*xy*_ becomes almost indiscernible even at a few Kelvin lower than *T*_N_ (blue marks). Such a characteristic temperature dependency is ubiquitously observed through the pressures of 1.4–2.2 GPa. The bottom figures of Fig. 2 show the field dependence of *σ*_*xx*_ as an important reference to the AHE state. At *T*_N_, the application of the field decreases *σ*_*xx*_ slightly like a typical magnetoresistance effect. However, the magnetoconductivity turns positive and shows a small hysteresis at lower temperatures. Particularly, in the narrow temperature region where the hysteresis of *σ*_*xy*_ is remarkably pronounced, the value of *σ*_*xx*_ ranges from ~700 Ω^−1^ cm^−1^ (at *P* = 1.4 GPa) to ~1000 Ω^−1^ cm^−1^ (at *P* = 2.2 GPa). Correspondingly, *σ*_*xy*_ increases from ~1.0 Ω^−1^ cm^−1^ to ~1.3 Ω^−1^ cm^−1^ with increasing pressure as seen in Fig. 2a–c. These obtained values of *σ*_*xx*_ and *σ*_*xy*_ reasonably fall onto the intrinsic AHE regime (AHE originating from the Berry phase mechanism)^24^ in which various families of ferromagnet such as a diluted magnetic semiconductor (Ga,Mn)As^25^ or a spinel-type chalcogenide CuCr_2_Se_4-*x*_Br_*x*_ with high critical temperature^26^ are categorized. With further lowering temperature, the hysteresis of *σ*_*xy*_ becomes nearly unobservable especially below ~200 Ω^−1^ cm^−1^ of *σ*_*xx*_, at which the charge gap starts to open^19,27^.

There are three main mechanisms of AHE which are widely accepted nowadays; the skew-scattering, the side-jump, and the intrinsic contribution^3^. The former two are attributed to a carrier scattering by crystalline disorders or impurities, and the last one is driven by Berry phase of a Bloch wave function. On the basis of prior experiments and microscopic theories^3^, it is natural to separate them by the transport lifetime. For this reason, we can safely rule out the skew-scattering contribution which basically is dominant in the high-conductivity regime (*σ*_*xx*_≥10^6^ Ω^−1^ cm^−1^). The rest mechanisms are the side-jump or the intrinsic; however, it is difficult to partition each other only according to the scaling rule between *σ*_*xx*_ and *σ*_*xy*_. We therefore pay our attention to the temperature dependency. As one can see in Fig. 2, the spontaneous part of *σ*_*xy*_ increases abruptly right below *T*_N_ and almost disappears at 1 K lower temperature, during which *σ*_*xx*_ decreases monotonically and smoothly. Since the side-jump mechanism originates from an impurity scattering, it can hardly explain the observed steep temperature dependence. Thus, we conclude that the intrinsic mechanism, which is sensitive to the electronic band structure, is most plausible for *σ*_*xy*_ observed in the course of the phase transition.

### Magnetization property of (Nd_0.5_Pr_0.5_)_2_Ir_2_O_7_

**Fig. 3:**
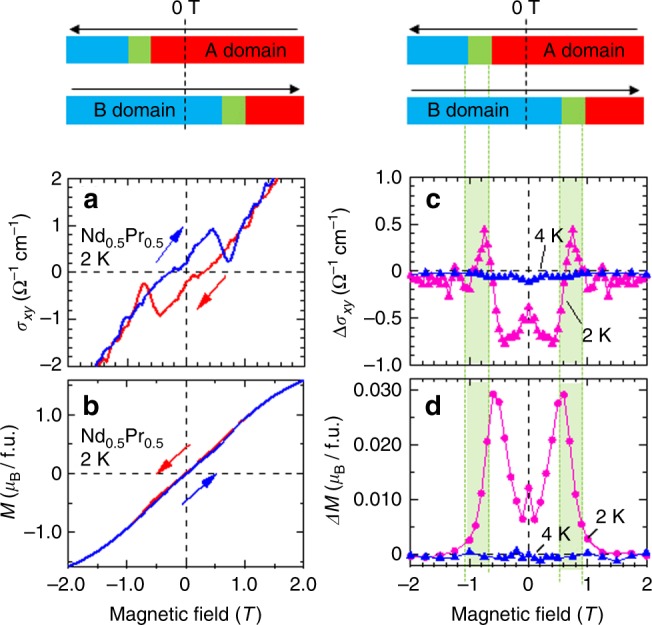
Hall conductivity and magnetization for (Nd_0.5_Pr_0.5_)_2_Ir_2_O_7_. Magnetic field dependence of **a** Hall conductivity and **b** magnetization for a magnetic field along [111] direction at 2 K. The blue (red) lines are Hall conductivity and magnetization on field-increasing (field-decreasing) process. The difference of **c** Hall conductivity and **d** magnetization between field-increasing and field-decreasing process. The magenta and blue denote 2 K (>*T*_N_) and 4 K (<*T*_N_), respectively. The top pictures show the domain states in each process. The red bars indicate A domain, blue ones are B domain, and green ones denote the domain flipping regions

Next, we examine the behavior of magnetization *M* which is anticipated to correlate with AHE. For the experiment we have grown single crystals of *R*-amalgamated (Nd_0.5_Pr_0.5_)_2_Ir_2_O_7_ (*T*_N_ = 3.8 K) which are large and qualified enough to measure both *M* and *σ*_*xy*_ reliably at ambient pressure without using the pressure cell. From the previous study [21] we note that the present *R* = Nd_0.5_Pr_0.5_ compound corresponds to the *R* = Nd compound under an effective pressure of *P* = 3.3 GPa (see Fig.1c). Figure 3a, b show the field dependence of *σ*_*xy*_ and *M* at 2 K. *σ*_*xy*_ exhibits a similar magnetic-field dependency to that for the undoped Nd_2_Ir_2_O_7_ under hydrostatic pressures except for the sharp close of the hysteresis loop at ±0.8 T. A hysteresis of *M* between field-increasing and field-decreasing process is barely visible in the same field range as well. To investigate the hysteresis behavior more closely, we show the difference of *σ*_*xy*_ (Δ*σ*_*xy*_) and *M* (Δ*M*) between the two processes in Fig. 3c, d, respectively. One can clearly see that both Δ*σ*_*xy*_ and Δ*M* exhibit significant field dependencies at 2 K (<*T*_N_) whereas they are almost zero at 4 K (>*T*_N_). Especially, the field dependency of Δ*σ*_*xy*_ is complex; starting from −0.5 Ω^−1^ cm^−1^ at 0 T, it decreases down to −0.8 Ω^−1^ cm^−1^ at ±0.6 T, abruptly jumps towards +0.5 Ω^−1^ cm^−1^ at ±0.8 T, and eventually goes to zero above ±1 T. To understand this behavior, we compare it with Δ*M*. Firstly it simply increases up to ~30 m*μ*_*B*_/f.u. with increasing field up to 0.6 T. This can be entirely accounted for in terms of the aligned AIAO single domain state that induces an asymmetric term of *M* ref. ^22^. Above 0.6 T, Δ*M* precipitously drops because of the switching of the magnetic domain. These behaviors are consistently observed in Nd_2_Ir_2_O_7_ as well^20^. Thus the domain state can be assigned as displayed in the top bars of Fig. 3. One might think that Δ*M* contains not only the contribution of Ir-5*d* moments but also that of *R*-ion 4 *f* moments. To check the contribution of the Ir-5*d* moment, we also measured *M* of Eu_2_Ir_2_O_7_ single crystal with non-magnetic *R* = Eu (see Supplementary Fig. 1). We have found that the value of the spontaneous *M* for *R* = Eu in the AIAO state is comparable with that for *R* = Nd_0.5_Pr_0.5_. It indicates that the Ir-5*d* moment is responsible for the observed Δ*M* perhaps because the single-ion anisotropy of *R*-4*f* moments is too strong to contribute to the near-zero-field *M*. On this basis, we conclude that the negative Δ*σ*_*xy*_ below 0.6 T, which appears to be correlated with Δ*M*, arises from the single-domain state, while the positive Δ*σ*_*xy*_ around 0.8 T corresponds to the domain switching. It is noteworthy that the magnitude of the present Δ*σ*_*xy*_ at 0 T is significantly large despite of the minimal value of Δ*M*. As mentioned above, the present system locates in the intrinsic Hall mechanism regime of the *σ*_*xx*_ vs. *σ*_*xy*_ realation. The representative ferromagnets in this regime such as (Ga,Mn)As or CuCr_2_Se_4-*x*_Br_*x*_ typically show *σ*_*xy*_~1–10 Ω^−1^ cm^−1^ and *σ*_*xx*_~1000 Ω^−1^ cm^−1^, which is nearly the same order of magnitude as that of the present system. However, as compared with the ferromagnetic magnetizations of these compounds, ~5 *μ*_*B*_ (Ga_0.8_Mn_0.2_As) or ~3 *μ*_*B*_ (CuCr_2_Se_4_) per magnetic atom, respectively, the spontaneous magnetization in the present system is astoundingly smaller by three orders of magnitude. It reminds us of the recent studies on the isostructural Pr_2_Ir_2_O_7_^27^ and the Heusler compounds Mn_3_*Z* (*Z* = Sn^28^ and Ge^29^) which exhibit large AHE with small magnetization. In the former compound, it is argued that the Pr-4*f* localized magnetic moment orders the ferromagnetic 2-in 2-out configuration below 0.3 K, inducing a spontaneous AHE via RKKY interaction between Pr-4*f* moments and Ir-5*d* itinerant electrons^27^. The latter compounds^28,29^ show exceptionally large AHE (*σ*_*xy*_~500 Ω^−1^ cm^−1^ with *M*~0.006 *μ*_*B*_/Mn) with non-colinear magnetic order. However, they are highly conductive metals with a large carrier number of ~1.9×10^22^ cm^−1^. Actually, the recent ARPES reveals that Mn_3_Sn has the large Fermi surface around *M* point as well as Weyl points which are ~60 meV above the Fermi energy^30^. On the other hand, the present compound Nd_2_Ir_2_O_7_ is predicted to host WPs right at the Fermi level^10^, which may enable us to directly probe an intriguing effect inherent to WPs such as chiral anomaly^31^.

Finally we mention the microscopic origin of the spontaneous magnetization in this material. The appearance of such a magnetization is not specific to the *R* = Nd or (Nd,Pr) compounds close to the WSM or band-touching SM state, but also observed for the AIAO state of the *R* = Eu compound (Supplementary Fig. 1) and *R* = Y^32-34^ with the well-defined charge gap^35^. At the moment, the microscopic origin of this weak ferromagnetism common to the AIAO state　of *R*_2_Ir_2_O_7_ is not clear, but hardly attributable to extrinsic origins, such as residual strain^23^ (see also Supplementary Note 1). On the basis of the magnetization measurement and a simple model simulation on polycrystals, reference^34^ suggests that the deviated valence of Ir ion creates non-magnetic defects of Ir^3+^, possibly inducing the ferromagnetism. However, it is somewhat different from the present case; a relatively small amount of defects in a single crystal, if any, may position randomly and the Hall current as well as the magnetization should be canceled out as a whole. It is more likely that the magnetic moments cant from the perfect AIAO pattern. This is consistent with the previous studies such as x-ray experiment revealing the *q* = 0 magnetic vector^36^. As shown in the following, the obtained values of both *M* and *σ*_*xy*_ are reproduced by our calculation which assumes the moment canting.

### Theoretical calculation of anomalous hall conductivity in weyl semimetal phase

One plausible origin of the observed AHE with vanishingly small *M* is a Berry curvature in the electronic band characteristic of the WSM state. On this assumption, we theoretically evaluate the magnitude of *σ*_*xy*_. We mimic the effect of magnetic moment on the Ir sites by an effective Zeeman field; the Hamiltonian has the form:

1 $${{H}} = H_{{\mathrm{Ir}}} - \mathop {\sum }\limits_i {\mathbf{m}}_i \cdot {\mathbf{\sigma }}_i.$$

**Fig. 4:**
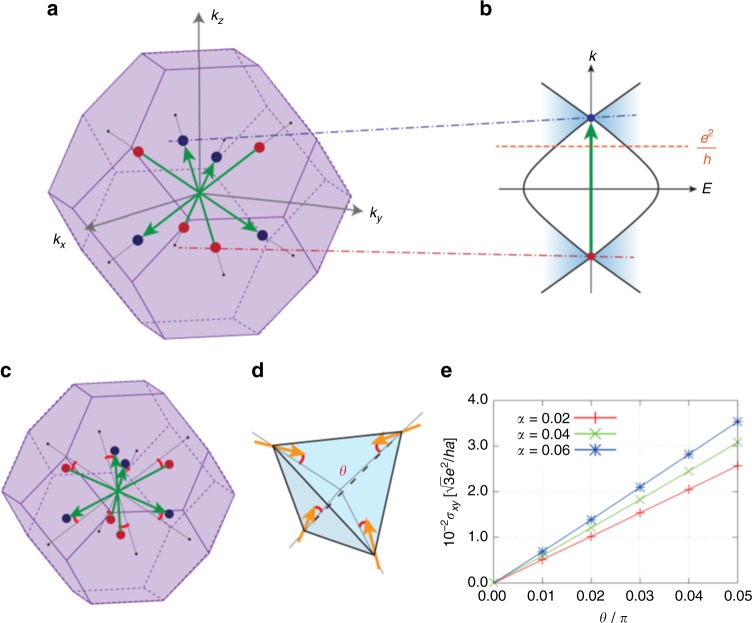
Theoretical description of Hall effect. **a** Schematic picture of the Brillouin zone and the Weyl points in the AIAO phase. The green arrows show the vector connecting the two Weyl points related by the spatial-inversion symmetry. The schematic figure of the band structure of the Weyl points is shown in **b** along the crystal momentum *k* along (111) direction. **c** The position of the Weyl points in the canted AIAO phase. The canted spin configuration is shown in **d**, and the Hall conductivity in the unit of $\sqrt 3 e^2/ha$ (*a* being the lattice constant) as a function of the canting angle *θ* is in **e**

Here, *H*_Ir_ is the single-particle Hamiltonian for the iridium *J*_eff_ = 1/2 electrons, **m**_*i*_ is the effective Zeeman field at the *i*-th site, and **σ**_*i*_ is the vector of Pauli matrices (see Methods). When |**m**_*i*_| = 0, a quadratic band touching exists at the Γ point^12,15^, which was recently confirmed experimentally^16^. When **m**_*i*_ is in the AIAO configuration, an infinitesimal |**m**_*i*_| splits the quadratic band touching into four pairs of WPs, each located along the eight 111 directions as shown in Fig. 4a. With further increasing |**m**_*i*_|, the WPs move away from the Γ point and eventually vanish by the pair annihilation at the *L* point on the zone boundary.

An interesting aspect of the WSM is that *σ*_*xy*_ sensitively reflects the position of the WPs as illustrated in Fig. 4b. Due to the property of WPs, the Chern number defined on each *k*_*z*_ plane ${\mathrm{C}}\left( {k_z} \right) = \frac{1}{{2\pi }}{\int} {\mathrm{d}{\it{k}}_x\mathrm{d}{\it{k}}_y{\it{b}}_z\left( {\mathbf{k}} \right)}$ changes by ±1 (*b*_*z*_(**k**) is the *z* component of the vector between a pair of WPs *b*(**k**)); in Fig. 4b, we illustrate an example where C(*k*_*z*_) = 0 outside the cone and C(*k*_*z*_) = *e*^2^/*h* inside. As *σ*_*xy*_ is proportional to the integral of C(*k*_*z*_) over *k*_*z*_, *σ*_*xy*_ increases when the distance between the two nodes increase and vice versa. In the case of pyrochlore iridates with the perfect AIAO order, the integral over C(*k*_*z*_) cancels out, resulting in zero *σ*_*xy*_. However, we find that the displacement of the WPs (Fig. 4c) associated with the canting of the spins (Fig. 4d) violates the cancellation.

In Fig. 4e, we show the result of *σ*_*xy*_ calculated using the Hamiltonian in Eq. (1) for three different *H*_Ir_ and fixed |**m**_*i*_| = 0.04 (Methods). To simulate the small *M* observed in the experiment, we cant the four moments toward the *z* axis as shown in Fig. 4d; the abscissa of Fig. 4e is the canting angle *θ*. This results in the shift of the WPs as schematically shown in Fig.4c and hence violates the cancellation of *σ*_*xy*_ As the consequence, *σ*_*xy*_ increases linearly with respect to the canting, and the canting of *θ*~10^−2^ rad gives the *σ*_*xy*_ value of 10^−2^ of the quantized value, $\sqrt 3 e^2/ha$ (*a* being the lattice constant). As the WSM phase of iridates is close to the boundary of MIT, the net moment induced by the canting of *θ*~10^−2^ rad is estimated about 10^−3^*μ*_*B*_/Ir (Methods). The calculated values of both *σ*_*xy*_ and *M* are consistent with the present experimental observations. These results imply the AHE in Nd_2_Ir_2_O_7_ as well as (Nd_1-*x*_Pr_*x*_)_2_Ir_2_O_7_, proximity to the magnetic phase boundaries, are likely to be a consequence of the Berry phase. Conversely, such a conspicuous zero-field Hall signal in the nearly antiferromagnetic state provides a compelling experimental evidence that the pyrochlore iridate under tuned conditions represent the WSM without time-reversal symmetry. The present study demonstrates that the topologically non-trivial feature gives rise to a salient Hall current with a minimal magnetization, pushing forward to the potential realization of next-generation dissipation-less devices.

## Methods

### Single crystal growth

Single crystals of Nd_2_Ir_2_O_7_ and its partially Pr-replaced (Nd_1-x_Pr_x_)_2_Ir_2_O_7_ were grown by the KF flux method. Firstly, mixtures of rare-earth oxides (Nd_2_O_3_ and Pr_6_O_11_) and iridate IrO_2_ were ground, pressed into pellets, and then heated at 1273 K for several days. Secondly, the obtained polycrystalline samples were ground again and mixed with KF flux in a ratio of 1:200. The mixtures are placed in a platinum crucible covered with a lid. The crucible was annealed at 1373 K for 3–5 h, and cooled down to 1123 K at a rate of 2 K/h. Finally, crystals were picked up from the KF residual flux by rinsing it out with distilled water. We obtained black octahedron-shaped single crystals which were characterized by x-ray diffraction.

### Transport and magnetization measurements

Transport, magnetization, and specific heat measurements were performed using Physical Property Measurement System (PPMS, Quantum Design). Resistivity and Hall conductivity was measured by a standard four-probe method with the current direction parallel to [110] crystalline direction and the magnetic field along [111] crystallographic direction. The Hall conductivity was deduced by the anti-symmetrization of the raw transverse signals perpendicular to the electric current. The pressure was applied by a piston-cylinder pressure cell for PPMS filled with Daphne 7474 oil as the pressure-transmitting medium. The pressure was determined by measuring the superconducting transition temperature of lead which was installed with samples.

### Theoretical analysis and models

An effective tight-binding model for *J*_eff_ = 1/2 orbitals of Ir electrons *H*_Ir_ is used for evaluation of the anomalous Hall conductivity^4^, of which the parameters of the nearest-neighbor hopping integrals follows that of a previous calculation used for Nd_2_Ir_2_O_7_^37^. Several different values for the ratio of nearest-neighbor and second-neighbor hopping integral *α* is considered, as shown in Fig. 4e. To simulate the effect of AIAO ordering and the small ferromagnetic moments observed in the experiment, a site-dependent Zeeman field is introduced as in Eq. (1). We set the magnitude of the Zeeman field to |**m**_*i*_| = 0.04, which is the parameter the Weyl nodes appear approximately at the center of Γ and *L* points. The calculation of the Hall conductivity was done using a superlattice of *N* = 4×192^3^ sites with periodic boundary condition. The size of the ferromagnetic moment **m**_FM_ is calculated using the same model, which were $\frac{{\left| {{\mathbf{m}}_{{\mathrm{FM}}}} \right|}}{{\mu _{{\mathrm{Ir}}}}} = 1.03 \times 10^{ - 3}$, 0.91 × 10^−3^, and 0.82 × 10^−3^, respectively for *α*= 0.02, 0.04, and 0.06, at *θ*= 0.01 rad. Here, *μ*_Ir_ is the size of magnetic moment for the *J*= 1/2 orbital of Ir^4+^ ions.

### Data availability

The data that support these findings are available from the corresponding authors on reasonable request.

## Electronic supplementary material

Supplementary Information

## References

[1] Hall EH (1881). On the “rotational coefficient” in nickel and cobalt. Philos. Mag..

[2] Karplus R, Luttinger JM (1954). Hall effect in ferromagnetics. Phys. Rev..

[3] Nagaosa N, Sinova J, Onoda S, MacDonald AH, Ong NP (2010). Anomalous Hall effect. Rev. Mod. Phys..

[4] Ye J (1999). Berry phase theory of the anomalous Hall effect: application to colossal magnetoresistance manganites. Phys. Rev. Lett..

[5] Shindo R, Nagaosa N (2001). Orbital ferromagnetism and anomalous Hall effect in antiferromagnets on the distorted fcc lattice. Phys. Rev. Lett..

[6] Taguchi Y, Oohara Y, Yoshizawa H, Nagaosa N, Tokura Y (2001). Spin chirality, Berry phase, and anomalous Hall effect in a Frustrated ferromagnet. Science.

[7] Onoda M, Nagaosa N (2002). Topological nature of anomalous Hall effect in ferromagnets. J. Phys. Soc. Jpn.

[8] Fang Z (2003). The anomalous Hall effect and magnetic monopoles in momentum space. Science.

[9] Yao Y (2004). First principles calculation of anomalous Hall conductivity in ferromagnetic bcc Fe. Phys. Rev. Lett..

[10] Wan X, Turner AM, Vishwanath A, Savrasov SY (2011). Topological semimetal and Fermi-arc surface states in the electronic structure of pyrochlore iridates. Phys. Rev. B.

[11] Yang. KY, Lu YM, Ran Y (2011). Quantum Hall effects in a Weyl semimetal: possible application in pyrochlore iridates. Phys. Rev. B.

[12] Witczak-Krempa W, Go A, Kim YB (2013). Pyrochlore electrons under pressure, heat, and field; shedding light on the iridates. Phys. Rev. B.

[13] Yamaji Y, Imada M (2014). Metallic interface emerging at magnetic domain wall of antiferromagnetic insulator: fate of extinct Weyl electrons. Phys. Rev. X.

[14] Young SM (2012). Dirac semimetal in three dimensions. Phys. Rev. Lett..

[15] Moon EG, Xu. C, Kim. YB, Balents L (2013). Non-Fermi-liquid and topological states with strong spin-orbit coupling. Phys. Rev. Lett..

[16] Kondo T (2015). Quadratic Fermi node in a 3D strongly correlated semimetal. Nat. Commun..

[17] Ueda K (2014). Anomalous domain-wall conductance in pyrochlore-type Nd_2_Ir_2_O_7_ on the verge of the metal-insulator transition. Phys. Rev. B.

[18] Ma EY (2015). Mobile metallic domain-walls in an all-in all-out magnetic insulator. Science.

[19] Nakayama M (2016). Slater to Mott crossover in the metal to insulator transition of Nd_2_Ir_2_O_7_. Phys. Rev. Lett..

[20] Ueda K (2015). Magnetic field-induced insulator-semimetal transition in a pyrochlore Nd_2_Ir_2_O_7_. Phys. Rev. Lett..

[21] Ueda K (2017). Magnetic-field induced multiple topological phases in pyrochlore iridates with Mott criticality. Nat. Commun..

[22] Arima Th (2013). Time-reversal symmetry breaking and consequent physical responses induced by all-in all-out type magnetic order on the pyrochlore lattice. J. Phys. Soc. Jpn.

[23] Fujita TC (2015). Odd-parity magnetoresistance in pyrochlore iridate thin films with broken time-reversal symmetry. Sci. Rep..

[24] Onoda S, Sugimoto N, Nagaosa N (2008). Quantum transport theory of anomalous electric, thermoelectric, and thermal Hall effects in ferromagnets. Phys. Rev. B.

[25] Chiba D, Nishitani Y, Matsukura F, Ohno H (2007). Properties of Ga1-xMnxAs with high Mn composition (x>0.1). Appl. Phys. Lett..

[26] Lee WL, Watauchi S, Miller VL, Cava RJ, Ong NP (2004). Dissipationless anomalous Hall current in the ferromagnetic spinel CuCr_2_Se_4-x_Br_x_. Science.

[27] Machida Y (2007). Unconventional anomalous Hall effect enhanced by a noncoplanar spin texture in the frustrated Kondo lattice Pr_2_Ir_2_O_7_. Phys. Rev. Lett..

[28] Nakatsuji S, Kiyohara N, Higo T (2015). Large anomalous Hall effect in a non-collinear antiferromagnet at room temperature. Nature.

[29] Nayak AK (2016). Large anomalous Hall effect driven by a nonvanishing Berry curvature in the noncolinear antiferromagnet Mn_3_Ge. Sci. Adv..

[30] Kuroda K (2017). Evidence for magnetic Weyl fermions in a correlated metal. Nat. Mater..

[31] Aji V (2012). Adler-Bell-Jackiw anomaly in Weyl semimetals: application to pyrochlore iridates. Phys. Rev. B.

[32] Shapiro MC (2012). Structure and magnetic properties of the pyrochlore iridate Y_2_Ir_2_O_7_. Phys. Rev. B.

[33] Zhu WK, Wang M, Seradjeh B, Yang Fengyuan, Zhang SX (2014). Enhanced weak ferromagnetism and conductivity in hole-doped pyrochlore iridate Y2Ir2O7. Phys. Rev. B.

[34] Yang WC (2017). Robust pinning of magnetic moments in pyrochlore iridates. Phys. Rev. B.

[35] Ueda K, Fujioka J, Tokura Y (2016). Variation of optical conductivity in the course of band-width controlled metal-insulator transitions in pyrochlore iridates. Phys. Rev. B.

[36] Sagayama H (2013). Determination of long-range all-in all-out ordering of Ir^4+^ moments in a pyrochlore iridate Eu_2_Ir_2_O_7_ by resonant x-ray diffraction. Phys. Rev. B.

[37] Tian Z (2016). Field-induced quantum metal-insulator transition in the pyrochlore iridate Nd_2_Ir_2_O_7_. Nat. Phys..

