# Supplementary material for: Spontaneous Hall effect in the Weyl semimetal candidate of all-in all-out pyrochlore iridate
Source: Nat Commun. 2018 Aug 2;9:3032. doi: 10.1038/s41467-018-05530-9 (PMC6072714; doi:10.1038/s41467-018-05530-9)
Supplement: Supplementary file 1 — Supplementary Information [file 41467_2018_5530_MOESM1_ESM.pdf]

Supplemental Information

Supplementary Note 1: The magnetic field and temperature dependence of magnetization in $Eu_2Ir_2O_7$.

Since *R*=Eu is a non-magnetic ion, $Eu_2Ir_2O_7$ is suitable to see the magnetic contribution of Ir-5*d* magnetic moments. To study the spontaneous term of magnetization for each domain state, we carried out magnetization measurements after magnetic field cooling along [111] crystalline direction and the opposite direction as schematically shown in Supplementary Fig. 1a; we first cool the sample with applying a field of 7 T down to 2 K, switch off the field, and then measure the magnetization with elevating the temperature. FiIt is to be noted here that the single-domain state cannot be realized by applying the magnetic field of several Tesla after the zero-field cooling in the R=Eu compound, contrary to the case of the *R*=Nd compound as described in the main text. The large Nd magnetic moments, which are coupled to the Ir-5*d* moments, can be easily controlled with the applied external field in the *R*=Nd compound. Thus, in the case of the *R*=Nd compound, one can tune the magnetic domain state of Ir-5d moments via *f*-*d* interaction by application of the magnetic field, e.g. along [111] direction, even at low temperatures.

Supplementary Figure 1b displays the magnetic field dependence of magnetization after the field cooling. One can clearly see the spontaneous component of $\pm 6 \times 10^{-3}\ \mu_{\mathrm{B}}/\mathrm{f.u.}$ at 0 T, the sign of which is opposite to each domain state. Supplementary Figure 1c and d show the temperature dependence of magnetization. The spontaneous magnetization monotonically decreases in parallel to the magnetic order parameter and vanishes above the transition temperature T_{N} at which the magnetization exhibits an anomaly in Supplementary Fig. 1c. The presence of the tiny spontaneous moment was reproducibly confirmed for any of *R*=Eu and (Nd,Pr) crystals showing the AIAO order.

Recently, Liang *et al.*[1] have reported on the possible presence of a hidden order developing from room temperature in $Eu_2Ir_2O_7$. This hidden order breaks the symmetry, which is very hard to detect and not identified as yet, and may produce this parasitic ferromagnetic moment when it is coupled to the AIAO order at lower temperatures.

Supplementary Figure 1

Temperature and magnetic field dependence of magnetization for $Eu_2Ir_2O_7$. (a) Measurement procedure of Supplementary Fig. 1b and 1d. Before the measurement, the sample was cooled down in an applied positive (negative) magnetic field to align A domain (B domain). (b) Magnetic field dependence of magnetization for A (red line) and B domain (blue line), respectively. The measurement was done at 2 K and [111] field direction after field cooling as shown in Supplementary Fig. 1a. (c) Temperature dependence of magnetization on the field cooling process. (d) Temperature dependence of magnetization for A domain (red marks) and B domain (blue marks). The measurement was carried out at 0 T on warming run after a field cooling as shown in Supplementary Fig. 1a.

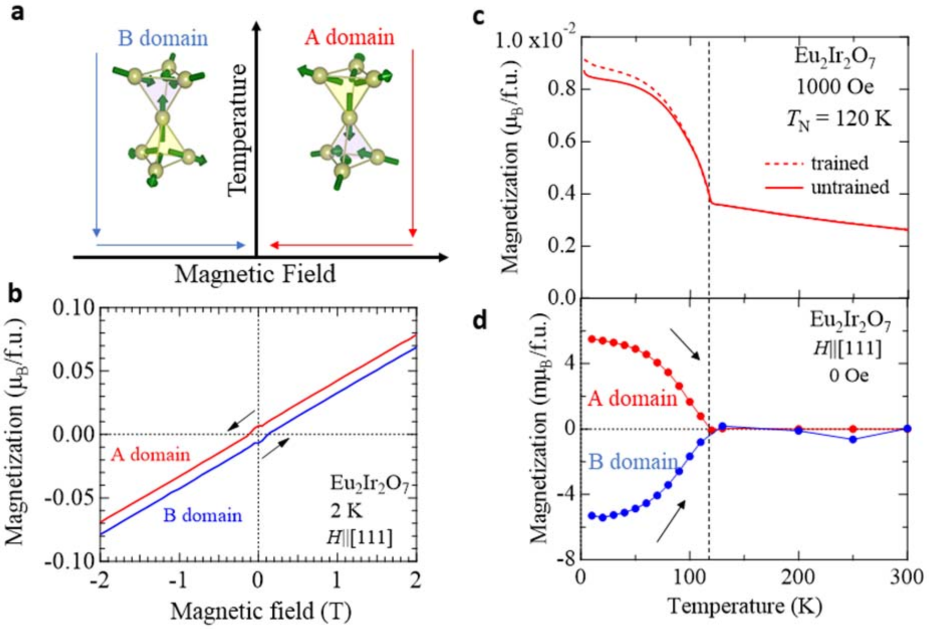

Supplementary References

[1] Tian, L., Hsieh, T. H., Ishikawa, J. J., Nakatsuji, S., Fu L., and Ong, N. P., Orthogonal magnetization and symmetry breaking in pyrochlore iridate $Eu_2Ir_2O_7$. *Nature Phys.* **13**, 599 (2017).
